# Genetic diversity of group A rotaviruses associated with repeated outbreaks of diarrhea in a farrow-to-finish farm: identification of a porcine rotavirus strain bearing a novel VP7 genotype, G26

**DOI:** 10.1186/1297-9716-42-112

**Published:** 2011-11-09

**Authors:** Ayako Miyazaki, Kazufumi Kuga, Tohru Suzuki, Mariko Kohmoto, Ken Katsuda, Hiroshi Tsunemitsu

**Affiliations:** 1 Viral Disease and Epidemiology Research Division, National Institute of Animal Health, 3-1-5 Kannondai, Tsukuba, Ibaraki 305-0856, Japan; 2 The United Graduate School of Veterinary Sciences, Gifu University, 1-1 Yanagido, Gifu 501-1193, Japan

## Abstract

Group A rotaviruses (GARs) are one of the most common causes of diarrhea in suckling pigs. Although a number of G and P genotypes have been identified in porcine GARs, few attempts have been made to study the molecular epidemiology of these viruses associated with diarrhea outbreaks within a farm over an extended period of time. Here, we investigated the molecular characteristics of GARs that caused four outbreaks of diarrhea among suckling pigs in a farrow-to-finish farm over the course of a year. G and P genotyping of GARs detected at each outbreak demonstrated genetic diversity in this farm as follows: G9P[23] was detected at the first outbreak, G9P[13]/[22] and G9P[23] at the second, G3P[7] at the third, and G9P[23], G5P[13]/[22], and P[7] combined with an untypeable G genotype at the fourth. Sequence analysis of the detected GARs revealed that such genetic diversity could have resulted not only from the introduction of new GAR strains, but also from gene reassortment between GAR strains within the farm. Further, the GAR strain carrying the untypeable G genotype was shown to be a novel porcine GAR bearing a new G26 genotype, as confirmed by the Rotavirus Classification Working Group.

## Introduction

Group A rotaviruses (GARs) are the most common etiological agent of severe diarrhea in infants and young children worldwide, as well as being a common cause of acute enteritis in young farm animals [1]. They are members of the *Rotavirus *genus, within the *Reoviridae *family, and their genome consists of 11 segments of double-stranded RNA (dsRNA) encased in a triple-layered capsid. These segments encode six structural (VP1-VP4, VP6, and VP7) and five or six non-structural proteins. Amongst these, the outer capsid proteins VP7 and VP4, which independently elicit the production of neutralization antibodies, define the G and P serotypes, and form the basis of a binomial nomenclature [1]. In recent years, the serotype classification system has been almost completely replaced by a genotyping classification system based on sequence differences of the respective gene segments [2,3]. Prior to the present study, at least 25 G genotypes and 33 P genotypes were identified in humans and animals [4,5]. Among them, at least 11 G genotypes (G1-G6, G8-G12) and 13 P genotypes (P[1], P[5]-P[8], P[11], P[13], P[19], P[13]/[22], P[23], P[26], P[27], and P[32]) have been described in pigs [6-11].

The zoonotic potential of animal GARs is a great concern [12]. GARs with unusual G genotypes commonly found in pigs and cattle have been detected in sporadic and epidemic cases of diarrhea in human populations [13-18]. A full genome-based genotyping system proposed by Matthijnssens et al. revealed the existence of reassortant strains and the close relationship between human and animal strains [2,3]. In addition, a number of new G and P genotypes have recently been identified in animals [4,5,7,19,20]. In light of these findings, animal GARs have come to be regarded as a potential reservoir for genetic diversity in human GARs, and studying them is therefore critical for understanding the evolution and ecology of human GARs.

Besides their zoonotic potential, as the most frequently detected enteropathogen associated with diarrhea among suckling pigs, GARs also pose an economic threat to the pig industry due to poor growth performance, and increased morbidity and mortality rates [21,22]. Given the present lack of a commercially available vaccine for GAR-associated diarrhea in Japan, the only means of prevention has been hygiene management. However, although effectively managing GAR infections requires an ecological understanding of GARs, few attempts have been made to examine the molecular epidemiology of porcine GARs associated with diarrhea within a farm over an extended length of time. We therefore investigated the genotypic characteristics of GARs causing repeated outbreaks of diarrhea among suckling pigs in a farrow-to-finish farm over the course of a year.

## Materials and methods

### Fecal samples

Outbreaks of epidemic diarrhea occurred among suckling pigs four times between February 2009 and March 2010 at a large farrow-to-finish farm with 4000 sows located in the Miyazaki prefecture, Japan. A total of 28 fecal samples (one sample per litter, 5 to 11 samples per outbreak) were collected from the rectum of diarrheal pigs that had not been treated with antibiotics. All animal experiments were approved by the Animal Ethical Committee and the Animal Care and Use Committee of National Institute of Animal Health.

### Bacteriological and parasitological examination

Bacteriological examinations were carried out for *Escherichia coli, Salmonella enterica*, and *Clostridium perfringens*, and parasitological examinations were carried out for *Coccidia *and *Cryptosporidium parvum*, as described previously [21].

### RNA extraction

Fecal specimens were diluted with Eagle's minimum essential medium to 10% suspensions and clarified by centrifugation at 1 500 × *g *for 10 min. The supernatant was collected and total RNA was extracted from 250 μL of the fecal suspensions using TRIzol LS (Invitrogen Corp., Carlsbad, CA, USA). Recovered total RNA was suspended in 50 μL of RNase/DNase-free water and stored at -20°C until use for reverse transcription-polymerase chain reaction (RT-PCR) and polyacrylamide gel electrophoresis (PAGE).

### RNA-PAGE

PAGE of the extracted RNA was performed with 7.5% precast gels (e-PAGEL; Atto Corp., Tokyo, Japan). The gels were stained using a Silver Stain Plus kit (Bio-Rad Laboratories, Hercules, CA, USA). GAR electropherotypes were determined by comparing the individual RNA migration patterns of genome segments on the gel [23].

### RT-PCR

The GAR VP7 gene and the VP8* fragment of the VP4 gene were amplified using a QIAGEN OneStep RT-PCR kit (Qiagen, Valencia, CA, USA) with primer pairs Beg9/End9 and Con2/Con3, respectively [24,25]. RT-PCR was conducted to detect group B and C rotaviruses (GBRs and GCRs), transmissible gastroenteritis virus, porcine epidemic diarrhea virus, and porcine sapovirus as described previously [26-30]. The amplicons were analyzed in 2% agarose gel electrophoresis and visualized by UV after ethidium bromide staining.

### DNA sequencing and genetic analysis

The amplicons of the GAR VP7 and VP4 genes were purified using MicroSpin S-400 HR Columns (GE Healthcare, Uppsala, Sweden). The purified PCR products were used as a template for sequencing on an Applied Biosystems 3100 automated DNA sequencer using Dye terminator cycle sequencing chemistry (Applied Biosystems, Foster City, CA, USA) and sequenced from both directions. To determine the complete coding region of the VP7 gene of a TJ4-1 strain, primers TJ4-1 494F (5'-GTA ACC CAA TGG ACA TTA CAC TG-3') and TJ4-1 368R (5'-CTA CTG AAA ATG ATG CGA TGT C-3') were also used as sequencing primers. The sequences were assembled, edited, and analyzed using MEGA 4 software [31]. The nucleotide sequences of the VP7 and VP4 genes from the detected GARs were compared with those of reference strains available in the GenBank. Multiple nucleotide sequence alignments were carried out using the CLUSTAL W algorithm. Genetic distances were calculated using the Kimura-2 correction parameter, and phylogenic dendrograms were constructed by the neighbor-joining method with 1 000 bootstrap replications [2].

### Nucleotide sequence accession numbers

The nucleotide sequences determined in this study have been submitted to GenBank under the following accession numbers: TJ1-1 (VP7:AB611688, VP4: AB621582); TJ2-1 (VP7:AB611689, VP4:AB621587); TJ2-2 (VP7:AB611690, VP4:AB621583); TJ3-2 (VP7:AB611692, VP4:AB621585); TJ4-1(VP7:AB605258, VP4:AB621586); TJ4-3 (VP4:AB611691, VP4:AB621584); TJ4-5 (VP7:AB611693, VP4:AB621588).

## Results

### Four outbreaks of GAR-associated diarrhea

Epidemic outbreaks of diarrhea affecting almost all suckling pigs born to 20% to 30% of lactating sows occurred in February, March, and May 2009, and March 2010. Common clinical signs included profuse watery diarrhea and dehydration lasting about one week. Mortality rates were less than 5%. Age and sow parity of the sampled pigs are summarized in Table 1. While no pattern was observed for the first two outbreaks, the third and fourth outbreaks mostly affected pigs less than seven days old that were born to gilts.

**Table 1:** Number, age, and sow parity of the sampled pigs at each of the four outbreaks of diarrhea on a large farrow-to-finish farm.

Outbreak	Month-year occurred	Number of sampled pigs	Range (median) of	
			
			Days of age	Sow parity
1	February 2009	11	4-10 (7)	1-7 (4)
2	March 2009	5	6-20 (9)	1-8 (6)
3	May 2009	5	2-5 (4)	1-1 (1)
4	March 2010	7	3-7 (5)	1-3 (1)

More than 80% of the samples collected at each outbreak were positive for GARs, as determined by RT-PCR targeting VP7, and are listed in Table 2. A small minority of samples contained other enteropathogens: GBR was detected in two samples at the first outbreak, *Isospora suis *and *Eimeria porci *were detected in one sample at the second outbreak, and GCR was detected in one sample at the fourth outbreak. These results indicate that GAR was the most common enteropathogen associated with the repeated outbreaks of diarrhea and most likely the cause as well.

**Table 2:** Electropherotype and combination of G and P genotypes of GAR strains detected in samples collected at each diarrheal outbreak.

Outbreak	Sample name	Electropherotype	Combination of G and P genotypes	
1	TJ1-1	eI	G9	P[23]
	TJ1-2	eI	G9	P[23]
	TJ1-3	eI	G9	nd
	TJ1-6	-^a)^	nd^b)^	nd
	TJ1-7	-	G9	P[23]
	TJ1-8	eI	G9	P[23]
	TJ1-9	eI	G9	nd
	TJ1-10	eI	G9	P[23]
	TJ1-11	eI	G9	P[23]

2	TJ2-1	eII	G9	P[13]/[22]
	TJ2-2	eI	G9	P[23]
	TJ2-3	eI	nd	nd
	TJ2-4	eII	nd	nd

3	TJ3-1	eIII	nd	nd
	TJ3-2	eIII	G3	P[7]
	TJ3-3	eIII	nd	nd
	TJ3-4	eIII	G3	P[7]
	TJ3-5	eIII	nd	nd

4	TJ4-1	eIV	G26^c)^	P[7]
	TJ4-2	eIV	nd	nd
	TJ4-3	eV	G9	P[23]
	TJ4-4	eV	nd	nd
	TJ4-5	eVI	G5	P[13]/[22]
	TJ4-6	eV	nd	nd

a) The sample was negative for GAR by RNA-PAGE.

b) The sample was positive for GAR by RT-PCR, but the nucleotide sequences could not be determined.

c) New VP7 (G) genotype determined in the present study.

### Electropherotypes of the detected GARs

**Figure 1:**
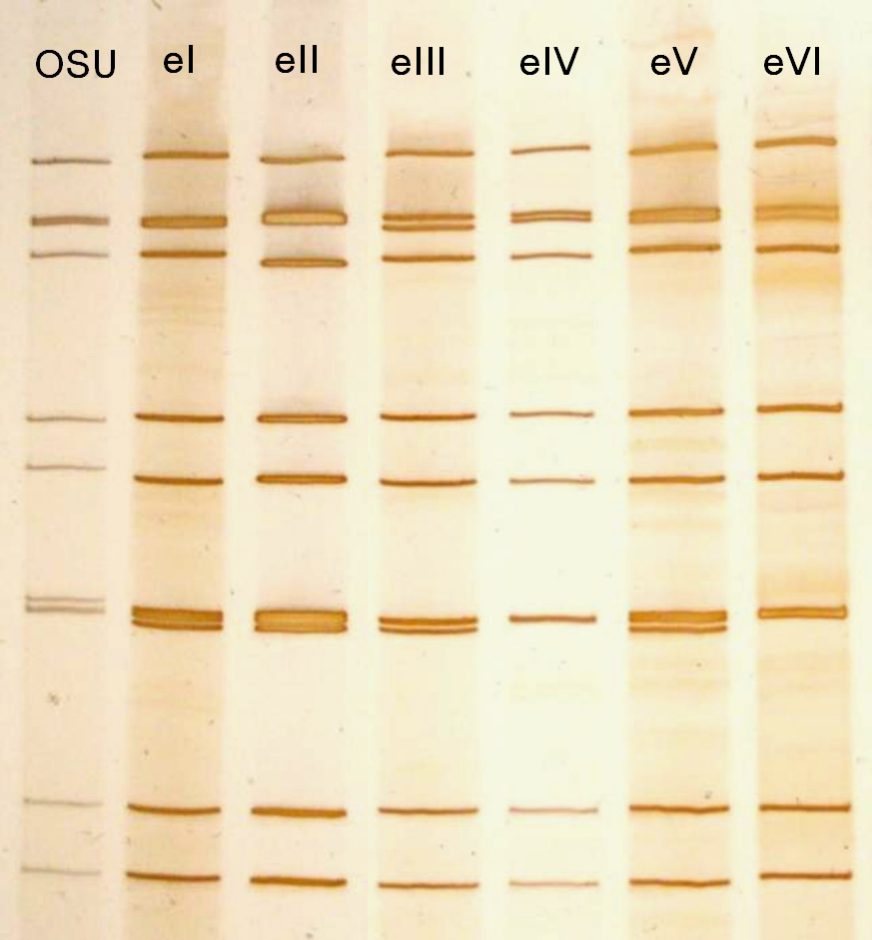
**Electropherotypes (eI to eVI) of GAR strains detected at each of the four diarrhea outbreaks in a farrow-to-finish farm from February 2009 to March 2010**. OSU is a reference porcine GAR strain with a long RNA migration pattern.

All 11 segments of GAR genomic RNA were visualized in 22 samples out of the 28 samples subjected to RNA-PAGE. These RNA migration patterns were classified into six electropherotypes designated eI to eVI (Figure 1). All electropherotypes displayed a long migration pattern resembling that of the porcine OSU strain (a reference porcine GAR strain). A different electropherotype was observed in each outbreak, as summarized in Table 2. RNA-PAGE found no samples to be infected with more than one GAR strain, although combined infection of GAR and GBR was observed in two samples (data not shown).

### Genetic analysis of GAR VP7 genes and identification of a novel G genotype

**Figure 2:**
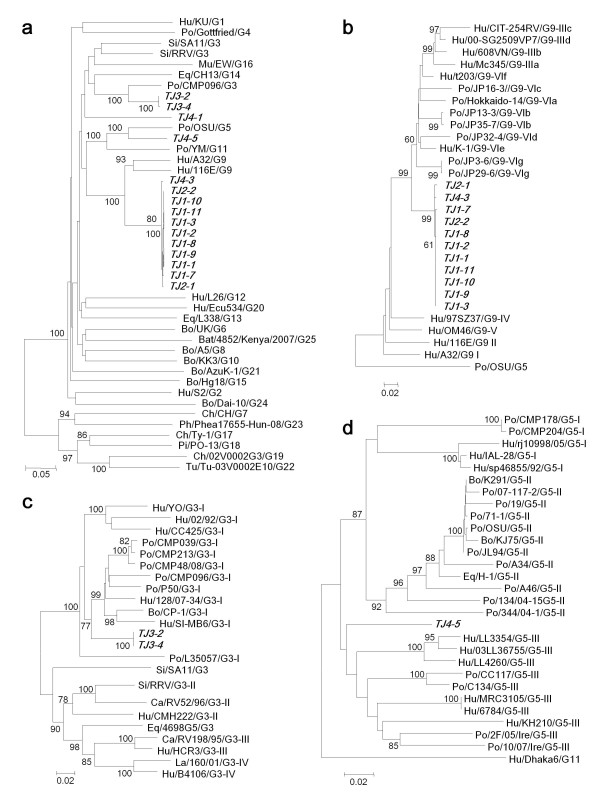
**Phylogenic trees based on the partial nucleotide sequences of VP7 genes from GAR strains identified in this study and those of representative or selected strains of each genotype**. (a) Phylogenic trees based on partial nucleotide sequences of the VP7 genes from GAR strains identified in this study and those of representative strains of each genotype. (b through d) Detailed phylogenic trees based on the partial nucleotide sequences of the VP7 genes from G9 (b), G3 (c), and G5 (d) strains identified in this study and also those of global origin. Bootstrap values above 60% are shown at the branch nodes. The strains identified in this study are highlighted in bold italic. The strains obtained using GenBank are shown with host species/strain name/genotype and lineage. Abbreviations: *Hu *human, *Po *porcine, *Bo *bovine, *Si *simian, *Mu *murine, *Eq *equine, *Ca *canine, *Bat *bat, *Ch *chicken, *Ph *pheasant, *Pi *pigeon, and *Tu *turkey. The GenBank accession numbers of the strains used in the phylogenic trees are listed in Additional file 1, Table S1.

The nucleotide sequences of the VP7 gene (933 bp, corresponding to nucleotides 69 through 1001 of the porcine G5 OSU strain's VP7 gene) were successfully determined in 15 samples. BLAST search analysis and phylogenic analysis of these VP7 genes with those of 25 established G genotypes led to classification of the 15 GAR strains into four genotypes: G9, G3, G5, and an untypeable G genotype (Figure 2a).

Eleven G9 strains were identified in this study, with eight strains detected at the first outbreak, two at the second, and one at the fourth (Table 2). A phylogenic tree based on a selection of human and animal G9 strains was generated as described by Collins et al. [32]. These eleven strains constituted a separate branch distantly related to human and porcine G9 strains comprising G9 lineages III and VI (Figure 2b). Nucleotide identities for the eleven strains were 99.4% to 100% compared to each other, 90.6% to 93.2% to the old Japanese porcine G9 isolates JP16-3, JP32-4, JP13-3, JP35-7, JP3-6, JP29-6, and Hokkaido-14 that belong to G9 lineage VI, and 87.2% to 93.8% to human and porcine G9 strains belonging to G9 lineages III and VI (Figure 2b and Additional file 1, Table S1).

The two G3 strains TJ3-1 and TJ3-2, both of which were identified at the third outbreak, constituted a separate branch distantly related to porcine G3 strains, comprising lineage I (Figure 2c) [33]. The nucleotide identities of their VP7 genes were 100% compared to each other, 87.6% to 91.6% to the porcine G3 strains comprising lineage I, 87.3% to 90.9% to human G3 strains comprising lineage I, and 79.1% to 82.4% to the G3 strains comprising other lineages (Figure 2c and Additional file 1, Table S1).

The G5 isolate TJ4-5, identified at the fourth outbreak, constituted a separate branch distantly related to human and porcine G5 strains comprising lineage III (Figure 2d) [34]. The nucleotide identities of its VP7 gene were 86.3% to 90.3% compared to the G5 strains comprising lineage III, 85.4% to 87.4% to lineage I, and 85.3% to 87.6% to lineage II (Figure 2d and Additional file 1, Table S1).

Phylogenic analysis revealed that the untypeable G genotype strain TJ4-1, identified at the fourth outbreak, did not cluster with any reference sequence from the group of 25 G genotypes (Figure 2a). In a BLAST search analysis, the nucleotide identities of its VP7 gene were 93.1% compared to 57vp7w, the uncommon human G3 strain identified in Thailand (736 bp, [GenBank:DQ674932]) [35], up to 80.5% to other G3 strains, and up to 78.8% to the strains of other G genotypes. We therefore characterized the TJ4-1 strain by analyzing the complete coding sequence of the VP7 gene, which turned out to be 981-bp long and included a predicted protein of 326 amino acids. We compared the nucleotide and deduced amino acid sequences with those from the reference strains of the 25 established G genotypes (Table 3) and found that the highest identity was to the G3 RRV strain (80.2% nucleotide identity and 87.5% amino acid identity), with lower nucleotide and amino acid identities to other strains ranging from 61.1% to 78.1%, and 57.2% to 85.5%, respectively. The Rotavirus Classification Working Group (RCWG) employs a cutoff value of 80% nucleotide identity to define a novel G genotype [3], and on submitting the sequence to the RCWG, it was subsequently assigned as a new G genotype: G26.

**Table 3:** Comparison of the nucleotide and amino acid sequence identities of the VP7 gene of TJ4-1 strain with those of 25 known G genotypes.

**Strain**^**a)**^	GenBank accession number	**Identity to TJ4-1 strain (%)**^**b)**^	
		
		Nucleotide	Amino acid
Hu/KU/G1	D16343	75.2	82.0
Hu/S2/G2	M11164	73.8	75.2
Si/RRV/G3	V01546	**80.2**	**87.5**
Si/SA11/G3	EU636932	77.7	85.3
Po/Gottfried/G4	X06759	72.9	74.6
Po/OSU/G5	X04613	76.9	81.3
Bo/UK/G6	X00896	74.7	82.0
Ch/Ch-2/G7	X56784	64.7	59.3
Bo/A5/G8	D01054	74.8	78.0
Hu/116E/G9	L14072	77.0	81.3
Bo/KK3/G10	D01056	74.6	80.7
Po/YM/G11	M23194	75.4	82.0
Hu/L26/G12	M58290	73.6	78.9
Eq/L338/G13	D13549	75.2	79.2
Eq/CH13/G14	D25229	78.1	81.0
Bo/Hg18/G15	AF237666	74.2	76.5
Mu/EW/G16	U08430	74.2	82.3
Tu/Ty-1/G17	S58166	64.7	60.6
Pi/PO-13/G18	D82979	64.7	57.5
Ch/02V0002G3/G19	FJ169861	63.1	57.2
Hu/Ecu534/G20	EU805775	74.6	81.0
Bo/AzuK-1/G21	AF454421	73.5	74.6
Tu/Tu-03V0002E10/G22	EU486973	61.1	58.3
Ph/Phea17655/Hun/08/G23	FN393056	64.5	58.5
Bo/Dai-10/G24	AB513837	71.4	73.2
Bat/KE4852/07/G25	GU983676	71.6	77.4

a) The reference strains are shown with host species/strain name/G genotype. Abbreviations for host species are shown in the legend of Figure 2.

b) The highest identity to the reference strain is shown in bold.

### Genetic analysis of the VP8* fragment of GAR VP4 genes

The nucleotide sequences of the VP8* fragment of the VP4 genes (774 bp, corresponding to nucleotides 70 through 843 of the porcine P[7] OSU strain's VP4 gene) were successfully determined in 13 of 15 samples in which the VP7 gene sequences had been previously determined. BLAST search analysis and phylogenic analysis of these VP4 genes with those of 33 established P genotypes led to classification of the 13 GAR strains into three genotypes: P[23], P[7], and P[13]/[22] (Table 2).

Eight P[23] strains were identified, with six strains detected at the first outbreak, one at the second, and one at the fourth (Table 2). These strains had nucleotide identities of 99.7% to 100% compared to each other, 91.0% to the porcine GUB71 strain identified in Japan, and 85.6% to 89.7% to other porcine P[23] strains (Additional file 2, Table S2).

**Figure 3:**
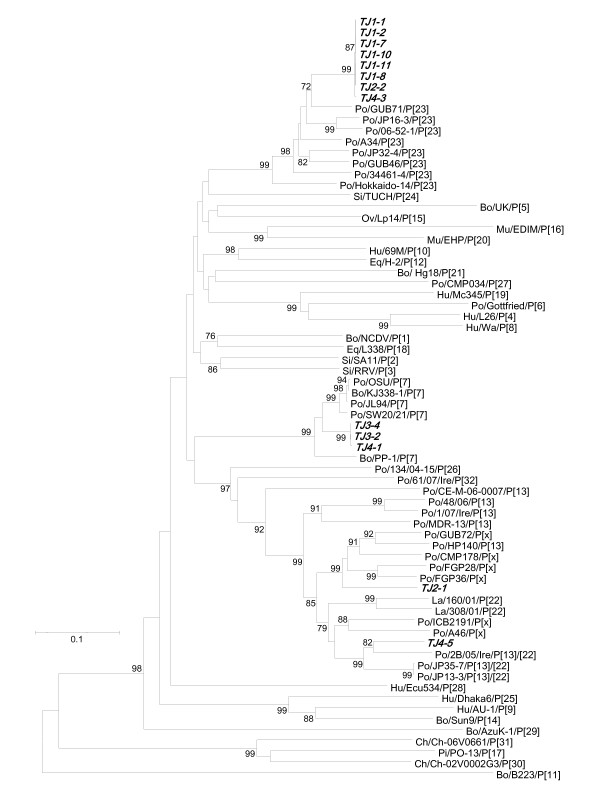
Phylogenic tree based on the partial nucleotide sequences of the VP8* fragment from GAR VP4 genes identified in this study and those of representative strains of each genotype. Bootstrap values above 60% are shown at the branch nodes. The strains identified in this study are highlighted in bold italic. The strains obtained using GenBank are shown with host species/strain name/genotype. Abbreviations: *Hu *human, *Po *porcine, *Bo *bovine, *Ov *ovine, *La *lapine, *Si *simian, *Mu *murine, *Eq *equine, *Ch *chicken, *Pi *pigeon, and *Tu *turkey. The GenBank accession numbers of the strains used in the phylogenic tree are listed in Additional file 2, Table S2.

Two P[7] strains (TJ3-1 and TJ3-2) were identified at the third outbreak and one (TJ4-1) at the fourth, having 99.7% to 100% sequence identities with each other. They were clustered in one branch related to the porcine OSU strain and in another to porcine and bovine P[7] strains (isolated in China and Korea respectively) with 86.4% to 94.5% nucleotide identity (Figure 3 and Additional file 2, Table S2).

In contrast, one P[13]/[22] strain identified at the second outbreak (TJ2-1) and one at the fourth (TJ4-5) showed low nucleotide identity (80.3%) with each other (Additional file 2, Table S2). The TJ2-1 strain belonged to a lineage comprised of porcine P[13] strains of Japanese origin (GUB72, FGP28 and FGP36) and global origin (CMP178 and HP140) with 83.3% to 84.6% nucleotide identity (Figure 3 and Additional file 2, Table S2). The TJ4-5 strain was clustered with the porcine P[13]/[22] strains of Ireland origin (2B/05/Ire) and Japanese origin (JP35-7 and JP13-3) with 89.0% nucleotide identity (Figure 3 and Additional file 2, Table S2).

### Combinations of G and P genotypes of GAR strains detected in the four outbreaks of diarrhea

Based on the sequence and phylogenic analysis of GAR strains detected among the four outbreaks, at least five combinations of G and P genotypes (G/P combinations) were identified: G9P[23] at the first outbreak, G9P[13]/[22] and G9P[23] at the second, G3P[7] at the third, and G26P[7], G9P[23], and G5P[13]/[22] at the fourth (Table 2).

Although the VP7 genes from the G9P[23] and G9P[13]/[22] strains were closely related to each other genetically, they combined with different P genotypes. Similarly, the VP4 genes from the G3P[7] and G26P[7] strains were highly homologous, yet they combined with different G genotypes.

## Discussion

Based on fecal analyses, we demonstrated that the cause behind repeated outbreaks of diarrhea among suckling pigs over a one-year period was primarily GAR infection. Sequence and phylogenic analysis of the VP7 and VP4 genes from detected GARs identified a spectrum of G and P genotypes, including G3, G5, G9, P[7], P[13]/[22], and P[23]. In addition, we identified the TJ4-1 strain, which contained a new VP7 genotype G26, as confirmed and assigned by the RCWG. Although the detected G and P genotypes (excluding G26) were common in pig populations worldwide [8,32,36,37], the strains identified in this study displayed marked genetic variation when compared to others around the world as well as older Japanese strains, suggesting genetic variability in GAR strains circulating in the pig population of Japan.

Several limitations to the present study warrant mention. Not only was the total number of samples low, but also, roughly half of them could not be successfully analyzed for either the VP7 or VP4 genes. However, despite these limitations, we found at least five different G/P combinations and six electropherotypes during the four outbreaks, indicating considerable genetic diversity of GAR strains within a farm continuing over one year. Co-circulation of different GAR strains in the same herd has also been reported in several studies: Collins et al. identified at least 2 to 6 GAR strains among asymptomatic pigs in each farm at a same time [32], and Barreiros et al. detected at least three strains during a diarrhea outbreak on a single swine farm [38]. While such genetic diversity in the same herd may have resulted from the introduction of new GAR strains, our results support the additional possibility of genetic reassortment between GAR strains within the herd. Indeed, the G9P[23] and G9P[13]/[22] strains contained almost identical G9 sequences, and the G3P[7] and G26P[7] strains had nearly identical P[7] sequences.

A previous study has shown that herds with GAR-associated diarrhea tend to be managed in an all-in all-out system and to have large numbers of sows [39]. Because diarrhea occurs in pigs when the oral challenge level of GAR exceeds the protective level of passive immunity acquired through colostrum and milk, the onset age and diarrhea severity are influenced by management practices that affect these factors, such as housing type, sanitation, and crate design [40]. By maintaining strict sanitation management and housing sows and gilts in separate units (about 4 000 sows were kept in 8 gestation and 14 farrowing barns), the farm in the present study might reduce the uniformity of exposure levels and immunity to GARs. The variation in affected pigs across the outbreaks appears to reflect the variation in the level of lactogenic immunity passed from sows to piglets (Table 1). Therefore, it cannot be ruled out that differing immunity levels among sows or gilts may be associated with the existence of the genetically diverse GAR strains and the repeated diarrhea outbreaks.

Based on accumulated evidence, it is clear that GARs can transfer between pigs and humans, making pigs a potential reservoir for the emergence of unusual or novel strains of human GARs [13-18]. In fact, the novel G26 strain we found was most similar to the human 57vp7w strain, which can also be considered to be a G26 strain. The 57vp7w strain was reported as a rare G3P[19] human strain identified in a clinical sample collected in Thailand between 2004 and 2006 [35]. Although the origin of G26 remains unclear, the VP4 sequence of 57vp7w [GenBank: DQ674935] is genetically closely related to those of porcine P[19] strains detected in Thailand [6]. Further, genetic analysis of several genome segments of the TJ4-1 strain other than VP4 and VP7 revealed that, except for the VP7 gene, this strain shares a similar genetic background with the porcine YM strain [Miyazaki A, in preparation]. Taken together, these findings suggest that 57vp7w may be a porcine-human reassortant or a porcine strain. Considering that the newly identified G26 has been detected in both pigs and humans, and the scarcity of animal GAR strains in the genetic database, further surveillance and epidemiological study on GARs in domestic animals will be needed to better understand the origin and circulation of human GARs.

Here, we demonstrated the genetic diversity of GARs associated with repeated outbreaks of diarrhea among suckling pigs within a farm over a one-year period. Such genetic diversity within a single farm might pose a challenge for developing effective methods of prevention against diarrhea caused by GAR infection.

## Competing interests

The authors declare that they have no competing interests.

## Authors' contributions

AM participated in designing the study, carried out the examination and molecular characterizations of GARs, and drafted the manuscript. HT conceived this study and participated in its design and coordination. KK and TS participated in the study design and coordination and carried out the virological examinations. MK and KK participated in designing the study and carried out the parasitological and bacteriological examinations. All authors read and approved the final manuscript.

## Supplementary Material

Additional file 1 **Table S1: Comparison of the VP7 genes of the 15 GAR strains identified in this study to those of reference and selected strains**. Comparison of the VP7 genes of the 15 GAR strains identified in this study to those of reference strains and a selection of G3, G5 and G9 strains. Click here for file

Additional file 2 Table S2: Comparison of the VP8* fragment of VP4 genes of the 13 GAR strains identified in this study to those of reference and selected strains. Comparison of the VP8* fragment of VP4 genes of the 13 GAR strains identified in this study to those of reference strains and a selection of P[7], P[23], and P[13]/[22] strains. Click here for file
